# A multidimensional exploration of the immediate physiological and psychological restorativeness of nature education for children in Giant Panda National Park

**DOI:** 10.3389/fpsyg.2026.1551296

**Published:** 2026-06-26

**Authors:** Ping Zhang, Yifei Zhao, Lilin Song, Ke Luo, Li Zhao, Fuqiang Chen, Mingze Chen, Rui Liu, Jinpeng Li, Yanbin Yang, Yuefeng He, Qingjie Zhang, Boya Wang, Limin Han, Yi Wang, Qin Yang, Xi Li

**Affiliations:** 1 College of Landscape Architecture, Sichuan Agricultural University, Chengdu, China; 2 Tangjiahe National Nature Reserve, Qingchuan, China; 3 College of Horticulture and Forestry Sciences, Huazhong Agricultural University, Wuhan, China; 4 Wenchuan Administration Station on Giant Panda National Park, Wenchuan, China; 5 Department of Forest and Resources Management, Faculty of Forestry, University of British Columbia, Vancouver, BC, Canada; 6 International Education School, Chengdu Agricultural College, Chengdu, China

**Keywords:** child health and development, computer vision model, Giant Panda National Park, immediate restorativeness, nature education

## Abstract

As the issue of “Nature-Deficit Disorder” among urban children becomes increasingly prominent, it is crucial to explore definite, scientific and systematic models of nature education. This aims to strengthen children’s connection with nature, promote their physical and mental health, enhance their self-awareness and social cognition, and improve their interpersonal relationships and decision-making skills. Using the methods of EEG (Electroencephalogram) monitoring, scale questionnaireing and facial recognition technology, this study shows how different forms of nature education impact children’s immediate physiological and psychological restorativeness with 35 child participants involved in direct, indirect and symbolic contact with nature for experiments. The study confirms that both direct and indirect contact with nature can enhance children’s concentration and relaxation, with no significant difference between them. Both forms of contact with nature can improve positive emotions and alleviate negative emotions for children. However, special attention to the form of direct contact with nature is still warranted. The study also preliminarily explored the feasibility of using Google Vision facial recognition technology in nature education. All the research is dedicated to helping with the issues arising from children’s “Nature-Deficit Disorder,” promoting children’s wellbeing, fostering sustainable and continuous protection of nature, and cultivating future citizens with a strong sense of social responsibility and good adaptive capacity.

## 1 Introduction

Existing research suggests that contact with nature can significantly benefit children’s holistic development, such as physical and mental health, sense of identity, social relationships, and levels of physical activity (van Dijk–Wesselius et al., 2018; Linzmayer and Halpenny, 2014). Contact with nature encourages pro-environmental behaviors and intentions in children (Martin et al., 2020; Cheng and Monroe, 2012), with even brief exposures yielding small but measurable responses (Beatley, 2011). Restorative environments refer to settings that can “promote (and not merely permit) restorativeness” of individuals’ resources (Hartig, 2004). A growing body of research demonstrates that restorative environments containing natural elements may offer multiple physiological and psychological benefits for children (Fyfe-Johnson et al., 2021). The positive emotional states children experience during nature contact indicate they derive restorative effects from their natural surroundings (Bagot et al., 2015). Concurrently, positive emotional responses can enhance outcomes in nature education, such as boosting learning motivation and stimulating greater curiosity for exploration. These benefits come specifically from nature contact, yet little research exists on the extent to which children experience restoration during this process. Given the potential role of nature contact in supporting or enhancing children’s physical and mental health, this gap is a critical one. However, due to urbanization, fast-paced lifestyles, guardians’ safety concerns and many other reasons, children’s contact with nature has decreased in both duration and frequency. Nature-Deficit Disorder (NDD), coined by Louv (2008), is a phrase to serve as a description of children’s cost of this phenomenon, which leads to a range of physical and mental health problems. NDD is a hypothesis rather than a clinically recognized disorder. The issue of NDD (Burdette and Whitaker, 2005; Clements, 2004; Juster et al., 2004) is becoming increasingly prominent, potentially leading to poor emotional regulation, risk assessment, environmental adaptation, a lack of curiosity about nature, and diminished adventurous spirit (Fu, 2021). It may also increase the likelihood of associated conditions such as sensory degradation, obesity, and depression (Huang, 2020; Yu and Du, 2022).

Nature education is defined as contacting with and experiencing natural elements, phenomena and cognitive processes within natural settings, aiming to foster acquaintance, understanding, and respect for nature, thereby cultivating ecological literacy and conservation awareness (Uzun and Keles, 2012). Nature education can deepen children’s understanding of global environmental issues (Çetinkaya, 2013), heighten conservation awareness, spark creativity, and foster positive environmental behaviors—ultimately promoting the sustainability of conservation efforts and their transfer across generations (Cetinkaya, 2013; Wang and Shi, 2020).

Nature education requires a natural environment as its foundation and demands sufficient space (Li, 2017). It utilizes outdoor venues such as schools, museums, parks, gardens, forests, as well as national parks and nature reserves (Yong, 2024) to rebuild children’s contacts with nature. Among them, national parks reservesent space as protected areas for managing and preserving special natural and cultural resourcesotected areas for managing and preservin Dudley et al., 2011), representative ecosystems of national or global importance, unique natural landscapes, and sites of rich scientific and cultural value (Tang, 2018), making them ideal locations for environmental education (Yao and Zhang, 2023).

For children to receive nature education, it is necessary for them to first have contact with nature. Children’s contact with nature can be categorized into three types: direct contact (physical interaction with nature in environments relatively free from human intervention and control), indirect contact (physical interaction but within more restricted, structured and human-managed settings), and symbolic contact (no physical interaction, with nature represented through virtual forms or electronic media) (Kahn and Kellert, 2002). Studies have noted that differences in the types of nature contact can result in varying behavioral outcomes. Previous studies have shown a positive relationship between nature-based education and children’s health (Sprague et al., 2020; Fyfe-Johnson et al., 2021). Exposure to greenspace may promote youth cognitive, socio-emotional and language (Sprague et al., 2022). Participation in environmental education activities (Otto and Pensini, 2017) and nature schools (Dopko et al., 2019) has been shown to have a significant positive impact on children’s pro-environmental behaviors and intentions, while living in greener communities is not necessarily associated with such behaviors (Collado et al., 2013; Martin et al., 2020). However, while the functions and practices of nature education for children are already quite diverse (Li et al., 2022; Yu et al., 2023; Li et al., 2023), empirical research on how different types and forms of contact with nature affect outcomes and underlying mechanisms remains limited.

Given the unique characteristics of children, the academic community commonly employs a mixed-method research approach, combining questionnaires and interviews. This approach involves using a “motherese” style of questioning during interviews to communicate with children and gather relevant information (Schaffer, 2004). Some studies have observed children attending forest schools, while conducting semi-structured interviews with school staff to indirectly reflect the characteristics of children’s activities (Harris, 2018). Additionally, some researchers have used children’s artwork created in national parks as a research tool to analyze their learning experiences and preferences regarding the environment (Labintah and Shinozaki, 2014). Also, some scholars have used scales and constructed models to assess adolescents’ emotional expression and learning outcomes in natural contexts (Dale et al., 2020). Other studies have used computer-based language analysis programs to examine children’s language samples, investigating the impact of natural environments on them (Novikova et al., 2024). Currently, the analyzed quantitative studies often rely on predefined and established questionnaire scales to measure emotional feedback, such as the Profile of Mood States (POMS) and the Positive and Negative Affective Scale (PANAS). Dopko et al. (2019) employed a shortened version of PANAS to measure children’s emotional responses to nature, further examining the potential benefits of nature experience on children’s mood and attitudes toward nature. PANAS enables the measurement of positive affect and negative affect, effectively assessing the emotional states of younger populations, including children and adolescents. However, children’s emotions can be fleeting and volatile. Due to limitations in cognitive abilities, emotional expression, and comprehension, relying solely on scales to measure children’s emotions may result in delayed responses, thereby compromising the accuracy of research findings. It is therefore essential to explore whether more objective, cost-effective technological methods can be used to study the interaction between children and their environment, along with children’s cognitive and emotional responses. Existing research has shown that human thoughts and emotions are reflected in facial expressions, which are the most significant factors in recognizing emotions. Facial expressions play a key role in understanding psychological states and determining appropriate responses, with visual factors contributing 55% to emotional understanding (Dalvi et al., 2021). Consequently, some scholars have proposed the use of visual cues for recognizing children’s emotions, finding that deep learning models in artificial intelligence (AI) can serve as highly efficient tools for emotional measurement. This technology has already been applied to online education platforms, where it is used to detect and analyze children’s focus, intentions, motivations and emotions, helping to develop better interactive solutions (Rathod et al., 2022). Therefore, the integration of AI for emotional measurement and nature education can provide a more objective and real-time analysis of children’s emotional experiences in natural environments. It can also offer personalized feedback to optimize nature education while enabling long-term tracking of children’s emotional changes at a low cost of human resource, supporting their psychological and social development. However, research on AI application to monitor and evaluate nature education benefits remains limited.

The original intention of the study is to better address the issue of children’s Nature-Deficit Disorder, develop a more scientific and efficient nature education practice model that targets children’s physiological and psychological health benefits, and provide educators and policymakers with feasible solutions for nature contact, thereby improving children’s physical and mental health, social adaptability and overall wellbeing. Based on this, the study aims to empirically explore the following questions:

1. What immediate physiological and psychological restorativeness of a multisensory direct-contact nature education in Giant Panda National Park?

2. Is there a difference in the immediate physiological and psychological restorative benefits for children before and after indirect forms of contact with nature, such as observation and creation? If so, how do these effects compare to the benefits of direct contact with nature?

3. How feasible is it to use AI-driven computer vision models (e.g., Google Vision) to analyze children’s emotional responses to nature education?

## 2 Materials and methods

### 2.1 Research subjects

For this study, 35 child volunteers, including 18 boys and 17 girls aged from 6 to 12, participated in the experiments. At first the participants shared similar levels of auditory-visual acuity and color recognition skills being in stable physical and mental health. One week before the experiment, all participants experienced relatively intense academic pressure for their age group and had not visited any parks or green spaces. Informed consent was obtained from all guardians including for human images. The study was approved by the Research Ethics Committee of Sichuan Agricultural University. To ensure the safety of the participants in outdoor activities and increase their identifiability, all of the children were equipped with standardized clothing.

### 2.2 Design of nature education experiments, experimental areas, and materials

The nature education experiments in this study consist of three types: direct contact through the “Ecological Observation Experience” in Giant Panda National Park (Experiment 1), indirect contact via “Scientific Production” in the park’s workshop (Experiment 2), and symbolic contact through a visually immersive experience of the “Flora and Fauna Species and Habitats of Giant Panda National Park” video (Experiment 3).

**FIGURE 1:**
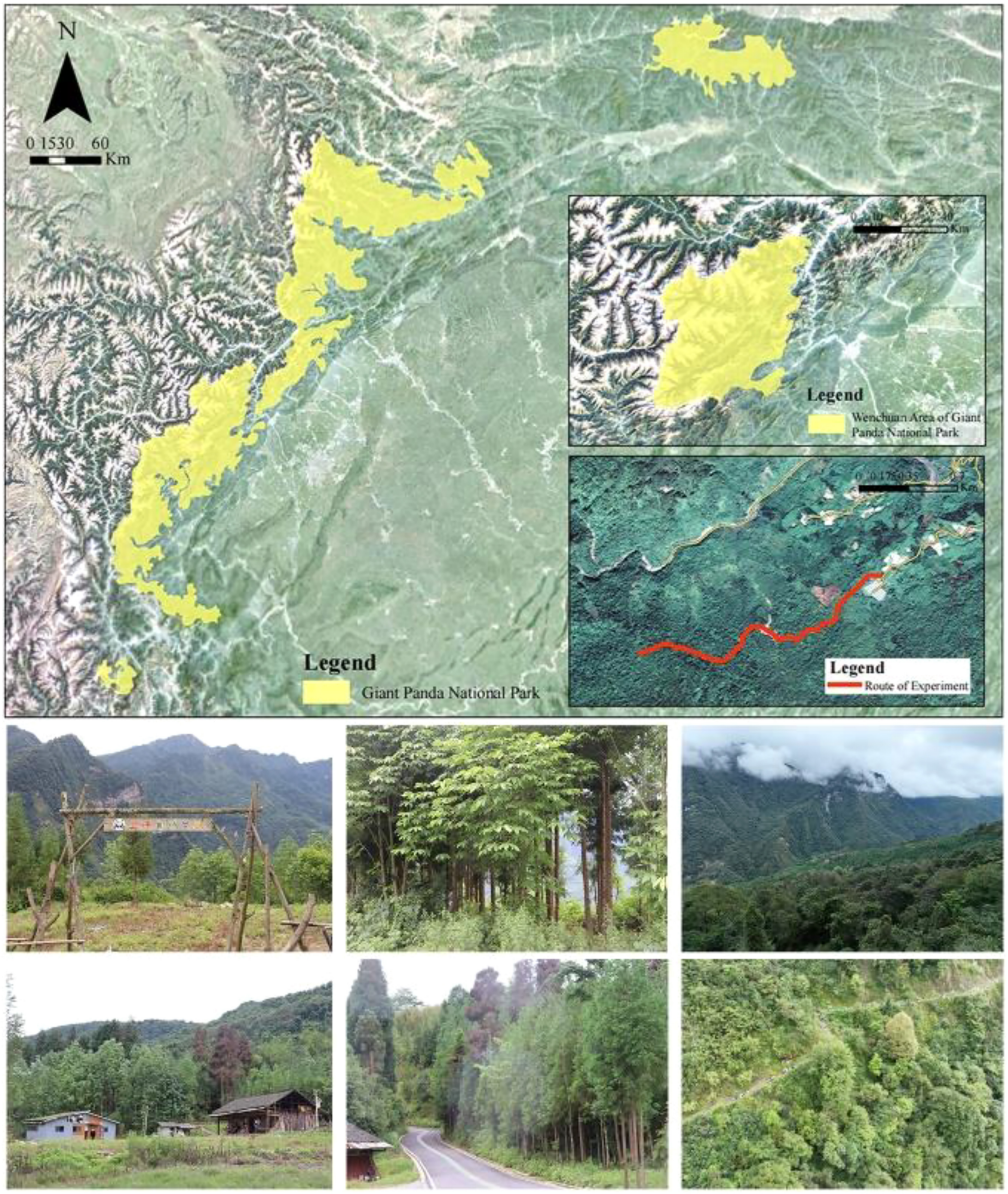
Area and Route for experiment 1. total route length is approximately 1,960 m.

In Experiment 1, participants followed a designated route within a section of Giant Panda National Park, a global biodiversity conservation gene bank, and a key unit of the UNESCO-designated “Sichuan Giant Panda Habitats” and “World Natural Heritage Site.” The area is rich in diverse plant species, featuring colorful pathways flanked by vegetation and scattered residences, forming a scenic landscape, ideal for nature education. The experiment route was based on input from the Park’s Management Station, patrol officers, and considerations of children’s physical capabilities and safety (Figure 1). Experiment 1 applied scientific instruments—such as a geological compass, fir cores, infrared cameras, and photosynthetically active radiation meters—to observe and identify plant species, morphology and habitats for flora and fauna. A ten-square-meter community quadrat was used to survey the ecosystem of the Park’s. Experiment 1 was conducted in a boundless pristine wilderness environmentrk’s. morphology and habitats for flora and fauna. A egetnce, utilized field exploration instruments to observe and experience the natural ecological environment, including habitats of flora and fauna.

Experiment 2 activities include making food for giant pandas (steamed buns) and using microscopes to observe and record their feces (qingtuan) as well as create plant specimens. Experiment 2 was conducted in an enclosed indoor environment (steamed buns) and using microscopes zed field exploration instruments tory instruments to observe and experience animal and plant specimens and their living habits.

**FIGURE 2:**
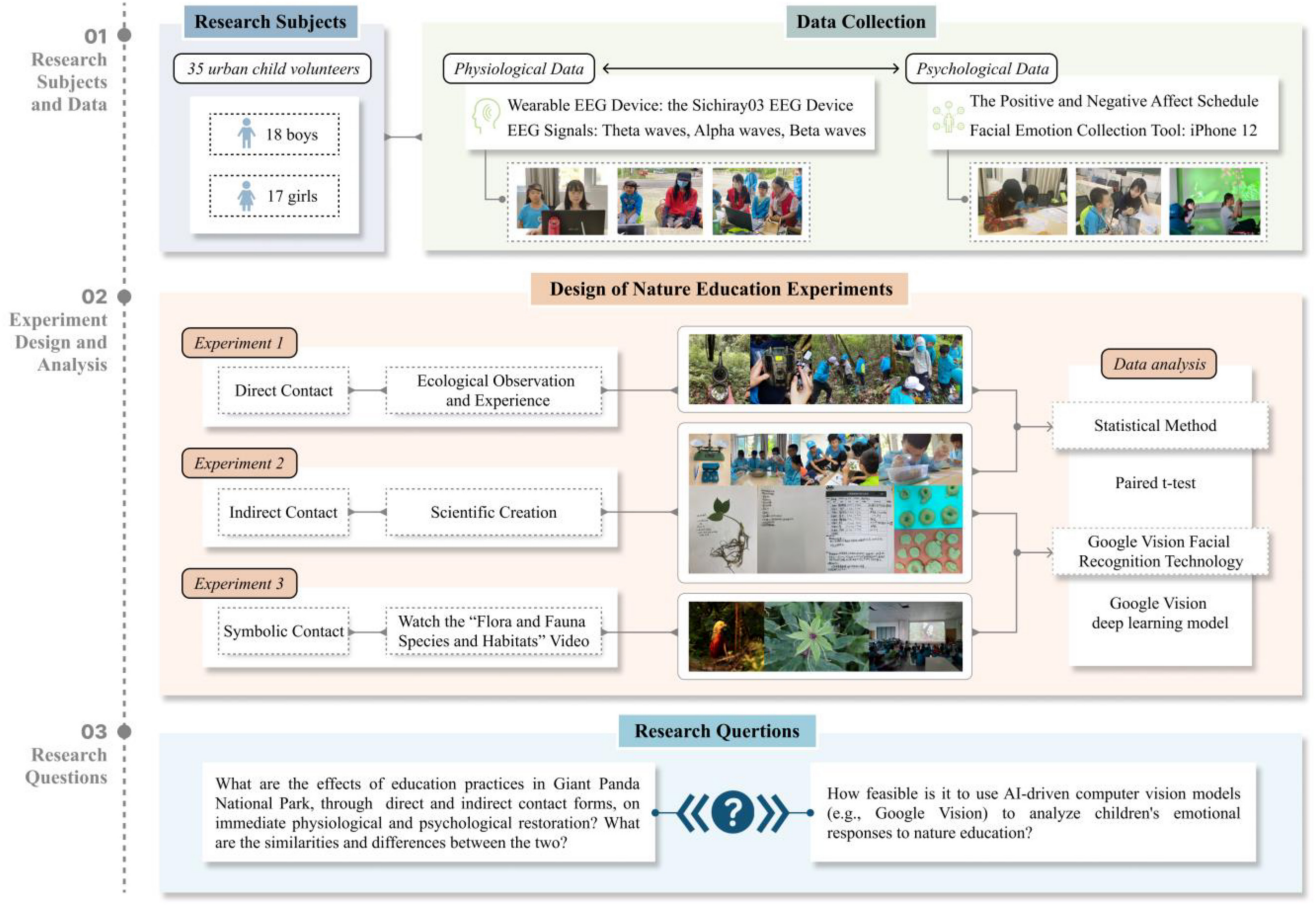
Research framework.

Experiment 3 activity was mainly watching a video on the “Flora and Fauna Species and Habitats of Giant Panda National Park.” Visual materials for the video were collected by the research team in the Park, using a Nikon Zfc with a Nikkor Z 24–120 mm f/4 S lens and an infrared camera (YEEINH888). Auditory stimuli included animal calls at a sound level between 35 and 38 dB (Aiwa 6228). The 300-s video was produced by XGIMI Technology Co., Ltd. using the XGIMI RS Pro3 projector with a resolution of 1,920 × 1,080, brightness of 2,500 CVIA, color gamut coverage of > 95% DCI-P3, > 99.9% Rec.709, a color accuracy of ΔE≈1, and onto a screen larger than 100 inches. Given the participants’ age, the device simulated a natural light spectrum (using Dual Light Super Mixed Light technology) to ensure eye comfort and avoid eye fatigue that could affect the experiment outcomes. The video content for Experiment 3 was educational and designed to avoid ethical or moral considerations related to animals, or storytelling, thus eliminating potential emotional interference (Figure 2).

### 2.3 Experimental methods

The study conducted EEG measurements for Experiments 1 and 2 with participants guided to complete the Positive and Negative Affect Schedule (PANAS). During Experiments 2 and 3, participants’ facial expressions were recorded, which were then analyzed using the Google Vision computer vision model.

#### 2.3.1 EEG measurement

Neuroscience has shown that EEG can reflect physiological responses of participants. The EEG signals measured in this study include Theta, Alpha and Beta waves. An increase in Theta-wave activity indicates enhanced relaxation, creativity, and learning ability (Schacter, 1977). The power of Alpha waves typically increases when individuals are in a state of wakefulness and physical relaxation (Fachner et al., 2013; Klimesch, 1999). Increase Beta wave activity is generally associated with alertness, while decreased Beta wave activity is related to drowsiness (Lee et al., 2014).

In this study, the Sichiray 03 EEG device was used for the experiment. By connecting wireless equipment and electrodes to the forehead, EEG data were displayed in real-time in the form of numerical values and waveforms, representing the participants’ levels of relaxation and focus during the experiment.

#### 2.3.2 Psychological measurement

Academic research has widely confirmed that as a predefined and established questionnaire scale reflecting emotions, PANAS possesses good validity and reliability. Therefore, this study employs PANAS to explore the emotional effects experienced by children after undergoing nature education, thereby reflecting restorative effects. The scale consists of various descriptive words and items divided into two main categories: positive emotions and negative emotions. It contains 20 items, each rated on a 5-point Likert scale, with 5 representing the highest level of positive emotion and 1 representing the lowest level of emotion. Previous studies have confirmed that this scale has good internal consistency reliability and construct validity (Watson et al., 1988; Medvedev et al., 2023) (see Supplementary Appendix).

Given children’s potential limitations in cognition, comprehension and expression, a “motherese” style of questioning was used for items on PANAS, such as “upset” and “alert.” Educational psychology professionals provided standardized training to the assistants of the research, ensuring that the guidance offered during the experiment had little influence on the willingness, authenticity or accuracy of the participants’ responses.

#### 2.3.3 Google vision facial recognition technology

This study applied the Google Vision deep learning model to facial emotion recognition for children. Google Vision is a pre-trained open-source model of Google Inc., supporting various applications such as text and facial detection, image labeling, and object recognition based on images. Previous research has demonstrated its effectiveness as a tool for studying human-environment interactions based on images. It features excellent image analysis capabilities, efficiently extracting and classifying emotions from facial images, such as happiness, sadness anger and surprise. In this study, 400 facial images were collected and the facial emotion collection tool is an iPhone 12, of which 200 images were collected respectively from Experiment 2 and Experiment 3. First, we applied Google Vision’s facial detection function to locate the faces within these 400 images. Second, the model conducted an in-depth analysis of the identified faces, detecting key features such as the eye, mouth and eyebrow, and then classified the emotions based on these features.

### 2.4 Experimental process

The experiment lasted 2 days, May 3–4, 2024. During these 2 days, the children’s experimental activities lasted for 2 h in the mornings, followed by 5 min in the evening on the second day. The time left was for free play and sleep, ensuring the children could have sufficient relaxation to prevent carryover effects. At 10:00 a.m. on May 3rd with temperature being 26°C ± 1°C, and humidity being 83%, 35 volunteers were taken to the national park by bus under the guidance of the assistants. Before the experiment, all the volunteers and their guardians had received relevant instructions, including the experiment rules and precautions. Volunteers and guardians completed an informed consent form about the wearing of the experimental apparatus, the monitoring of the experimental data, and the consent to the use of human images. Upon arrival at the park, the participants rested in a seated position for 10 min. Then they completed the EEG and psychological measurements through PANAS. Since the participants’ cognitive and comprehension capabilities varied due to their age, the experiment did not include a stress-increase test. Instead, the research team monitored the participants’ learning stress levels over the previous week to ensure consistent physiological and psychological stress level before the experiment. Subsequently, under the guidance of an assistant with a PhD in ecology, participants entered the national park to engage in Experiment 1 activities, which lasted for 2 h. After completing the activities, their EEG data were collected again, and they completed PANAS once again.

At 10:00 a.m. on the morning of May 4th, the experiment assistants gathered the participants at the nature education workshop, in which the temperature was controlled at 26°C, to carry out Experiment 2 with the same procedure. During the experiment, the assistants were divided into groups to collect facial image data, with each assistant collecting data every 72 s without interfering with participants’ activities.

**FIGURE 3:**
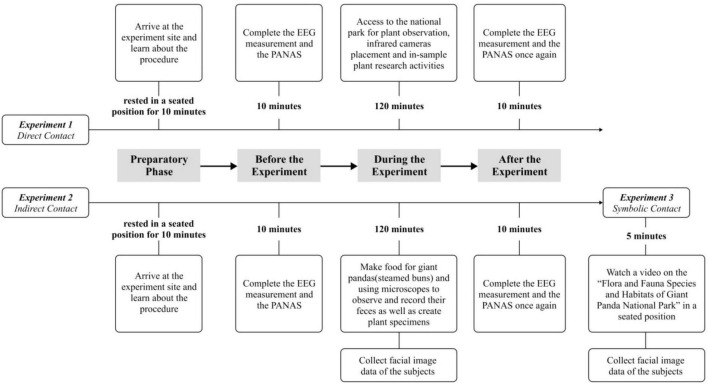
Experimental process diagram. The experimental methods in this study could not achieve full consistency for the following reasons. First, Experiment 1, conducted in the outdoor environment of the national park, encountered uncontrollable factors such as changing natural light conditions, dense vegetation obstructing views and spatial limitations in certain areas where children moved more extensively (e.g., narrow or steep parts of the trail). These factors made it extremely challenging to collect high-quality facial images, which could introduce biases in the facial recognition results. Therefore, facial image data collection was not performed during Experiment 1. Second, given that physiological and psychological data collection (including various scales and questionnaires) needed to be conducted repeatedly before and after each experiment, and Experiment 3 involved only a brief period of video watching, the study aimed to avoid causing restlessness or resistance among younger children or those with low tolerance, which could compromise data accuracy. Consequently, only facial image data were collected during Experiment 3, with no EEG or PANAS data collected before or after Experiment 3.

At 7:00 p.m. on the evening of May 4th, the participants were gathered again and taken to the nature education workshop, in which the temperature was at 26°C ± 1°C. After a 10-min rest, participants watched the “Flora and Fauna Species and Habitats of Giant Panda National Park” video in a seated position for 5 min. During the video viewing, three staff members collected facial image data, with each group of staff collecting data every 3 s without disturbing the viewing experience (Figure 3).

### 2.5 Statistical analysis

The study applied SPSS 26.0 for statistical analysis. First, the Shapiro-Wilk test was conducted to verify data normality (all indicators: *p* > 0.05). Subsequently, a paired *t*-test was used for the following analyses: comparing the significance of changes in EEG, PANAS positive and negative emotion mean values before and after Experiment 1 and Experiment 2, as well as assessing the significance of changes in the mean values of EEG Theta, Alpha, and Beta waves after Experiments 1 and 2.

## 3 Results

### 3.1 Physiological results

#### 3.1.1 Experiment 1: paired *t*-test results for EEG values before and after the direct-contact nature education experiment

**FIGURE 4:**
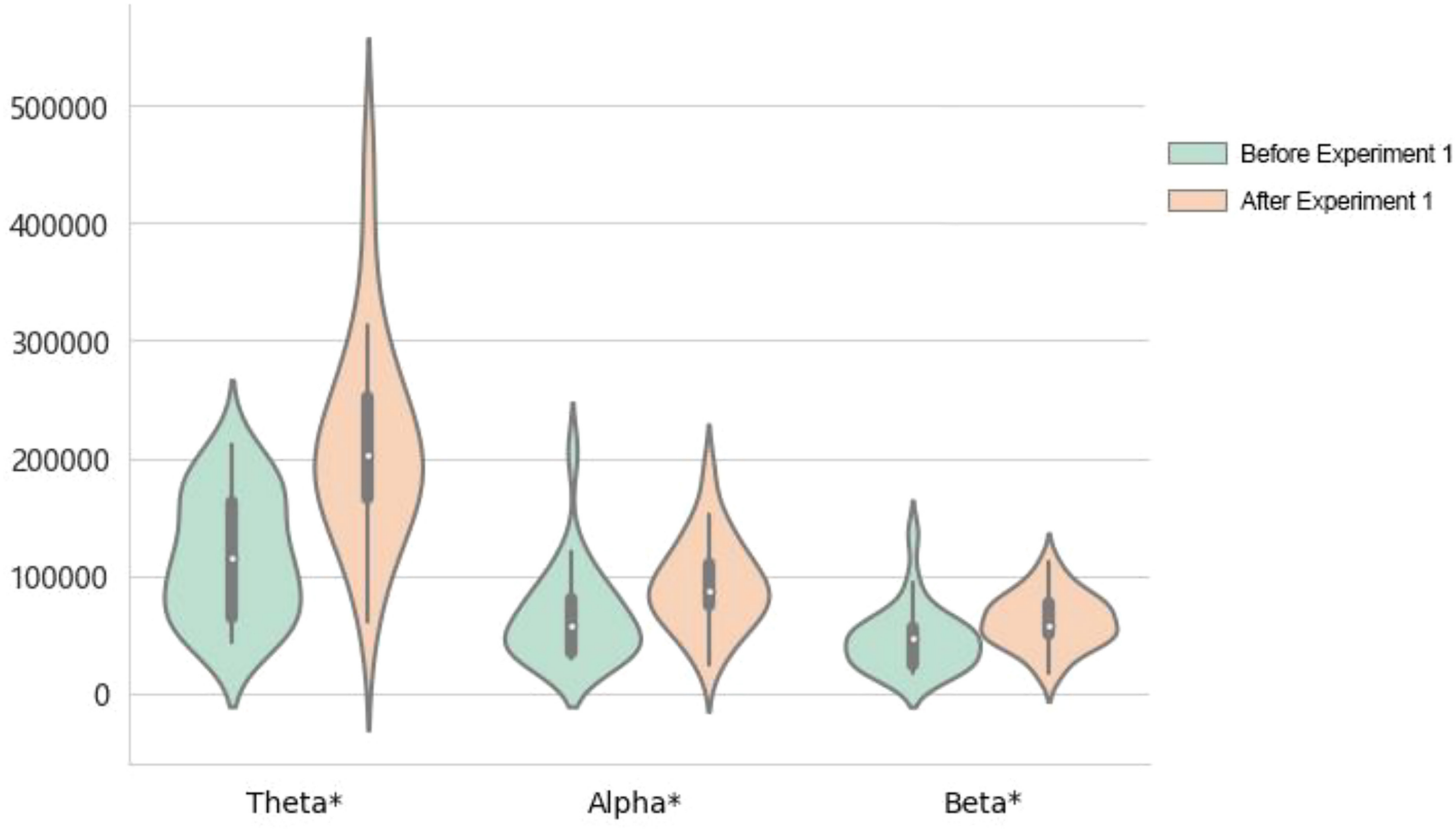
Paired *t*-test results for EEG values before and after Experiment 1.

The paired *t*-test results showed that, compared with pre-experiment values, the EEG values of Theta, Alpha, and Beta waves significantly increased following the completion of Experiment 1. The average Theta wave value increased by 97,597.09 (by 84.0%, *P* = 0.000), the average Alpha wave value increased by 26,995.30 (by 41.3%, *P* = 0.024), and the average Beta wave value increased by 15,920.65 (by 34.5%, *P* = 0.050). The post-experiment median and primary distributions of Theta, Alpha, and Beta wave values also exhibited a significant upward trend, indicating a notable improvement in the children’s relaxation and focus levels (Figure 4).

#### 3.1.2 Experiment 2: paired *t*-test results for EEG values before and after the indirect-contact nature education experiment

**FIGURE 5:**
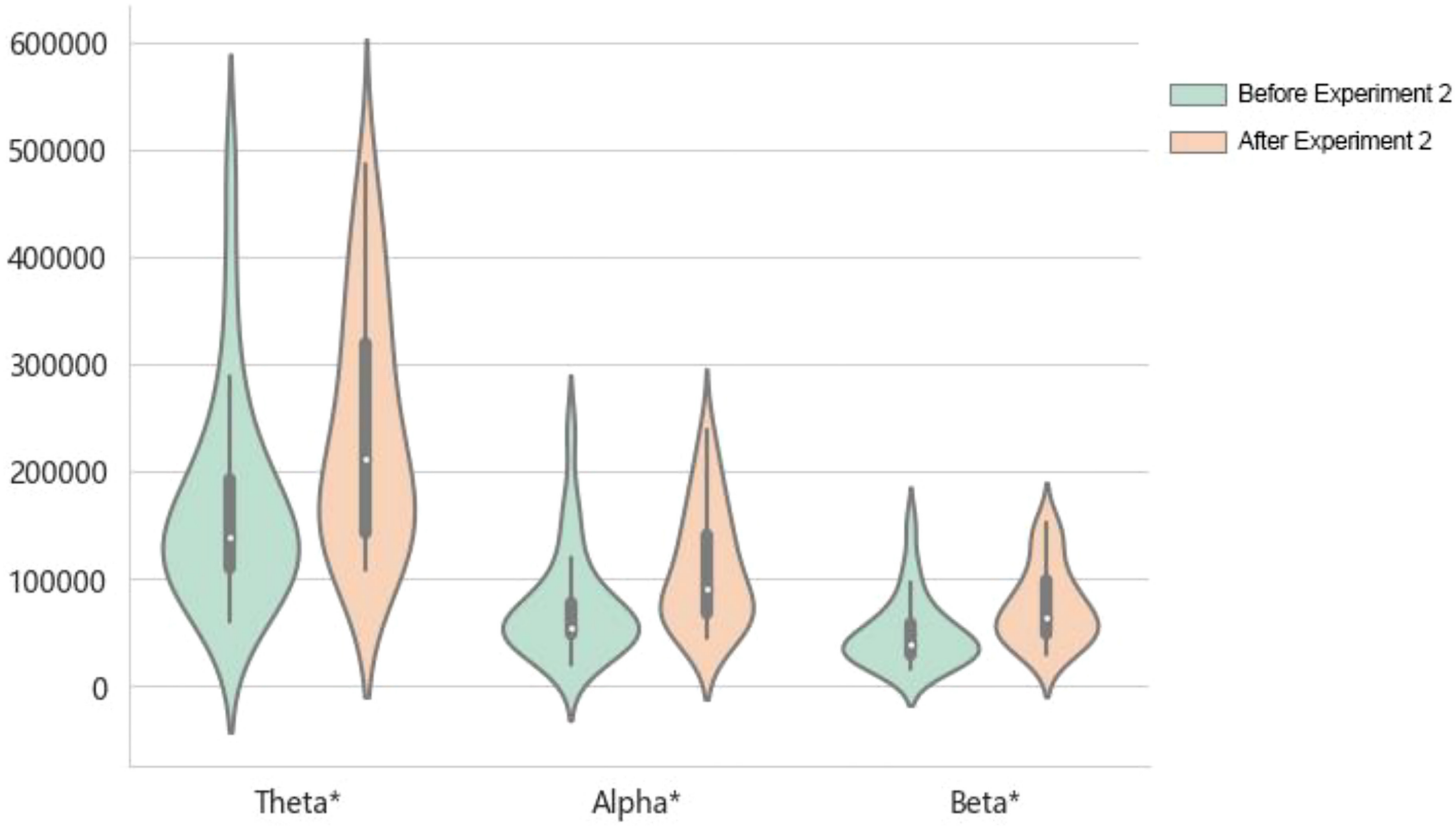
Paired *t*-test results for EEG values before and after Experiment 2.

The results showed a significant increase in the values of Theta, Alpha, and Beta waves. Specifically, the mean Theta wave value increased by 68,512.91 (by 10.9%, *P* = 0.007), the mean Alpha wave value increased by 35,879.30 (by 19.0%, *P* = 0.003), and the mean Beta wave value increased by 25,817.64 (by 20.5%, *P* = 0.002). The post-experiment median and primary distributions of Theta, Alpha, and Beta wave values also exhibited a significant upward trend, indicating a notable improvement in children’s relaxation and concentration levels (Figure 5).

#### 3.1.3 Paired *t*-test results for EEG values after experiment 1 and experiment 2

**FIGURE 6:**
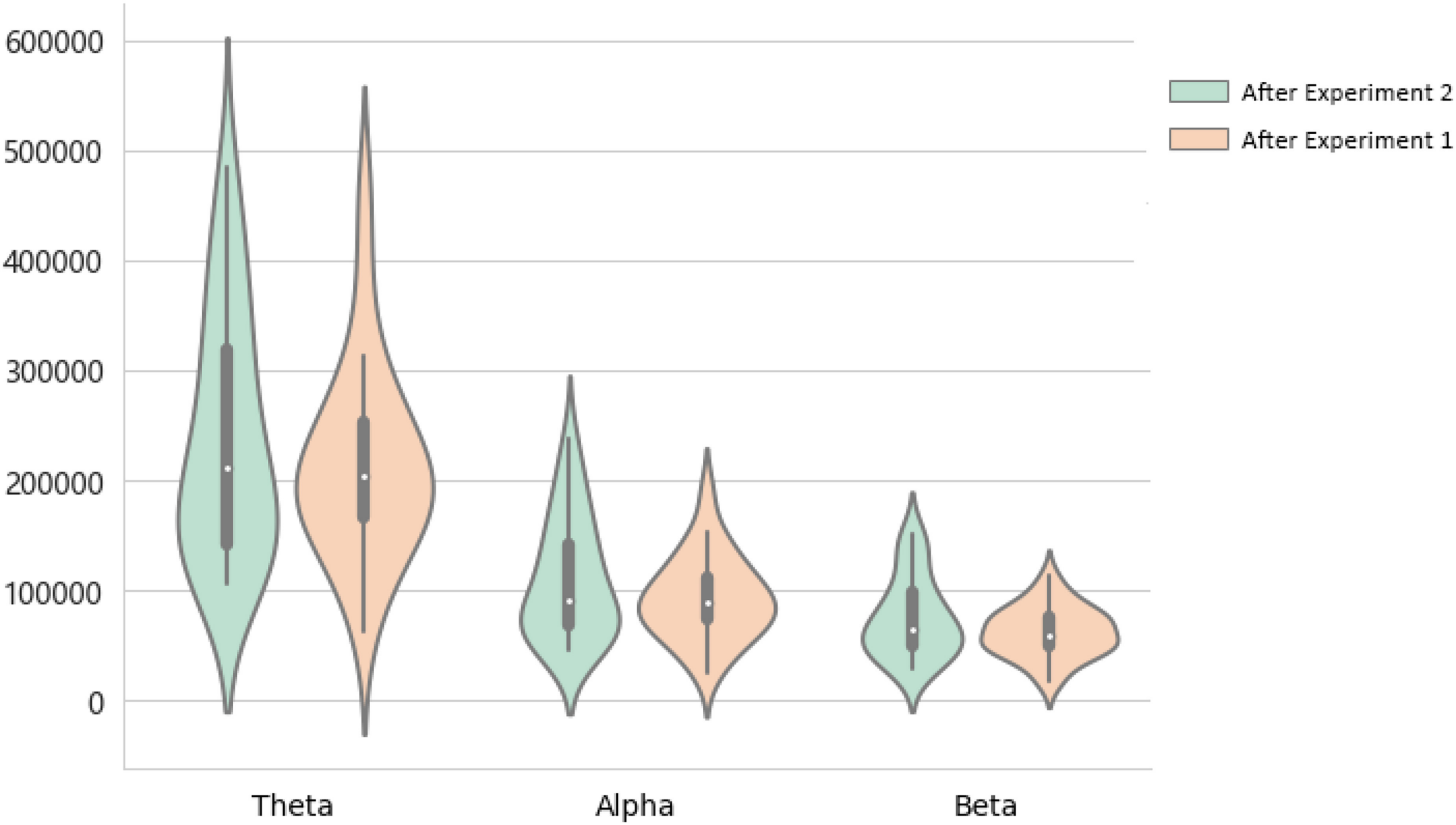
Comparison of EEG values after Experiment 2 and Experiment 1 (paired *t*-test results).

No significant differences were observed between Experiment 1 and Experiment 2 (Figure 6). Although the increase in Theta wave values was higher after Experiment 1 compared with Experiment 2 (an average increase of 97,597.09 for Experiment 1 versus 68,512.91 for Experiment 2), the average increases in Alpha and Beta wave values were slightly lower after Experiment 1 than after Experiment 2 (an average Alpha increase of 26,995.30 for Experiment 1 versus 35,879.30 for Experiment 2; an average Beta increase of 15,920.65 for Experiment 1 versus 25,817.64 for Experiment 2). Furthermore, the median values of Theta, Alpha, and Beta waves had no significant difference between the two experiments, though the overall distribution range of the three waves was broader after Experiment 1 than after Experiment 2.

**FIGURE 7:**
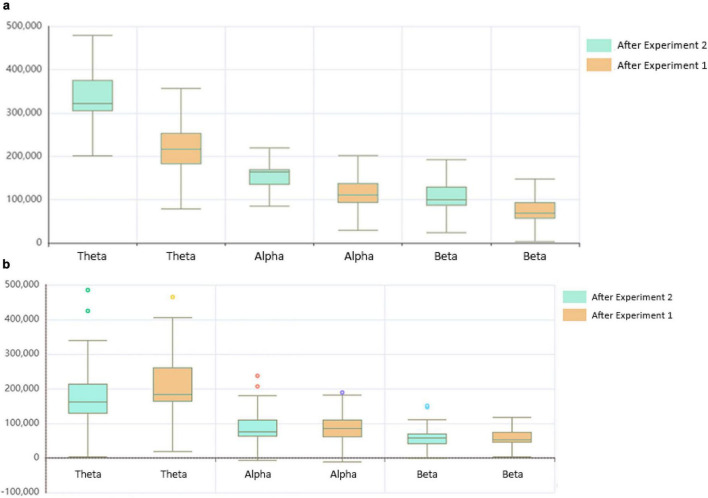
**(a)** Comparison of EEG values (Theta, Alpha, Beta) for the 11 participants after experiment 2 and experiment 1. **(b)** Comparison of EEG values (Theta, Alpha, Beta waves) for the 24 participants after experiment 2 and experiment 1.

These results suggested that both direct and indirect nature contact could significantly improved children’s relaxation and concentration, with no significant difference between the two forms in terms of impact on EEG values. However, Experiment 1 seemed to have a slight advantage over Experiment 2 in enhancing overall relaxation and attention levels. Further analysis revealed that 11 participants exhibited higher EEG values (Theta, Alpha, Beta) after Experiment 2 compared with Experiment 1 (Figure 7a). This group included 7 boys (2 aged 6, 2 aged from 8 to 9, and 3 aged from 11 to 12) and 4 girls (2 aged 8 and 2 aged from 10 to 11). The remaining 24 participants displayed at least one EEG value lower after Experiment 2 compared with Experiment 1 (Figure 7b).

### 3.2 Psychological results

#### 3.2.1 Experiment 1: PANAS results before and after the direct-contact nature education experiment

The results of PANAS before and after Experiment 1 indicated that, with the exception of the “guilty” indicator among negative emotions, which showed no significant change, all positive emotion indicators significantly increased, while all negative emotion indicators significantly decreased. This suggested that direct contact with nature could significantly reduce children’s negative emotions and enhance their positive emotions (Tables 1, 2).

**TABLE 1:** Paired *t*-test results for positive emotions before and after experiment 1, containing means, standard deviations, standard error of mean, 95% confidence intervals, *t*-value, degree of freedom and significance.

Pair	Emotion	Paired differences					*t*	df	Sig. (2-tailed)
		M	SD	SEM	95% CI				
					Lower	Upper			
Pair 1	Interested (before) —Interested (after)	–0.692	1.050	0.206	–1.116	–0.268	–3.363	34	0.002**
Pair 2	Excited (before) —Excited (after)	–1.115	1.306	0.256	–1.643	–0.588	–4.354	34	0.000***
Pair 3	Strong (before) —Strong (after)	–1.000	0.980	0.192	–1.396	-0.604	–5.204	34	0.000***
Pair 4	Enthusiastic (before) —Enthusiastic (after)	–0.769	1.070	0.210	–1.201	–0.337	–3.666	34	0.001***
Pair 5	Proud (before) —Proud (after)	–0.769	1.107	0.217	–1.216	–0.322	–3.544	34	0.002**
Pair 6	Alert (before) —Alert (after)	–0.769	1.243	0.244	0.267	1.271	3.156	34	0.004**
Pair 7	Inspired (before) —Inspired (after)	–0.846	1.156	0.227	–1.313	–0.379	–3.734	34	0.001***
Pair 8	Determined (before) —Determined (after)	–0.808	1.059	0.208	–1.235	–0.380	–3.889	34	0.001***
Pair 9	Attentive (before) —Attentive (after)	–0.462	0.948	0.186	0.079	0.844	2.483	34	0.020*
Pair 10	Active (before) —Active (after)	–1.192	1.327	0.260	–1.728	–0.656	–4.581	34	0.000***

*N* = 35.

*p < 0.05,

**p < 0.01,

***p < 0.001.

**TABLE 2:** Paired *t*-test results for negative emotions before and after Experiment 1, containing means, standard deviations, standard error of mean, 95% confidence intervals, *t*-value, degree of freedom and significance.

Pair	Emotion	Paired differences					*t*	df	Sig. (2-tailed)
		M	SD	SEM	95% CI				
					Lower	Upper			
Pair 1	Distressed (before) —Distressed (after)	1.115	1.143	0.224	0.654	1.577	4.976	34	0.000***
Pair 2	Upset (before) —Upset (after)	0.885	1.071	0.210	0.452	1.317	4.213	34	0.000***
Pair 3	Guilty (before) —Guilty (after)	0.192	0.749	0.147	–0.110	0.495	1.309	34	0.203
Pair 4	Scared (before) —Scared (after)	0.846	1.008	0.198	0.439	1.253	4.282	34	0.000***
Pair 5	Hostile (before) —Hostile (after)	0.462	0.761	0.149	0.154	0.769	3.094	34	0.005**
Pair 6	Irritable (before) —Irritable (after)	0.692	1.158	0.227	0.224	1.160	3.048	34	0.005**
Pair 7	Ashamed (before) —Ashamed (after)	0.615	1.525	0.299	–0.001	1.231	2.057	34	0.050*
Pair 8	Nervous (before) —Nervous (after)	0.654	0.936	0.183	0.276	1.032	3.563	34	0.002**
Pair 9	Jittery (before) —Jittery (after)	0.577	0.902	0.177	0.213	0.941	3.261	34	0.003**
Pair 10	Afraid (before) —Afraid (after)	0.885	1.275	0.250	0.370	1.400	3.537	34	0.002**

*N* = 35.

*p < 0.05,

**p < 0.01,

***p < 0.001.

#### 3.2.2 Experiment 2: PANAS results before and after the indirect-contact nature education experiment

The results from PANAS before and after Experiment 2 indicated that most positive emotions significantly increased, while most negative emotions significantly decreased. Exceptions included “attentive” among positive emotions, as well as “guilty” and “jittery” among negative emotions, which showed no significant change. These findings suggested that indirect contact with nature could also lead to noticeable improvement in children’s negative emotions and significant enhancement of positive emotions (Tables 3, 4).

**TABLE 3:** Paired *t*-test results for positive emotions before and after Experiment 2, containing means, standard deviations, standard error of mean, 95% confidence intervals, *t*-value, degree of freedom and significance.

Pair	Emotion	Paired differences					*t*	df	Sig. (2-tailed)
		M	SD	SEM	95% CI				
					Lower	Upper			
Pair 1	Interested (before) —Interested (after)	–0.577	0.945	0.185	–0.959	–0.195	–3.112	34	0.005**
Pair 2	Excited (before) —Excited (after)	–0.885	1.423	0.279	–1.460	–0.310	–3.169	34	0.004**
Pair 3	Strong (before) —Strong (after)	–0.769	1.366	0.268	–1.321	–0.218	–2.872	34	0.008**
Pair 4	Enthusiastic (before) —Enthusiastic (after)	–0.654	1.164	0.228	–1.124	–0.184	–2.864	34	0.008**
Pair 5	Proud (before) —Proud (after)	–0.500	0.990	0.194	–0.900	–0.100	–2.575	34	0.016*
Pair 6	Alert (before) —Alert (after)	–0.538	1.174	0.230	0.064	1.013	2.339	34	0.028*
Pair 7	Inspired (before) —Inspired (after)	–0.692	0.928	0.182	–1.067	–0.317	–3.803	34	0.001***
Pair 8	Determined (before) —Determined (after)	–0.462	0.905	0.177	–0.827	–0.096	–2.601	34	0.015*
Pair 9	Attentive (before) —attentive (after)	–0.308	0.884	0.173	–0.665	0.049	–1.775	34	0.088
Pair 10	Active (before) —Active (after)	–0.692	1.644	0.322	–1.356	-0.028	–2.148	34	0.042*

*N* = 35.

*p < 0.05,

**p < 0.01,

***p < 0.001.

**TABLE 4:** Paired *t*-test results for negative emotions before and after the Experiment 2, containing means, standard deviations, standard error of mean, 95% confidence intervals, *t*-value, degree of freedom and significance.

Pair	Emotion	Paired differences					*t*	df	Sig. (2-tailed)
		M	SD	SEM	95% CI				
					Lower	Upper			
Pair 1	Distressed (before) —Distressed (after)	1.000	1.327	0.260	0.464	1.536	3.844	34	0.001***
Pair 2	Upset (before) —Upset (after)	0.654	1.263	0.248	0.144	1.164	2.640	34	0.014*
Pair 3	Guilty (before) —Guilty (after)	–0.192	1.234	0.242	–0.691	0.306	–0.795	34	0.434
Pair 4	Scared (before) —Scared (after)	0.615	1.169	0.229	0.143	1.087	2.685	34	0.013*
Pair 5	Hostile (before) —Hostile (after)	0.462	0.989	0.194	0.062	0.861	2.379	34	0.025*
Pair 6	Irritable (before) —Irritable (after)	0.654	1.263	0.248	0.144	1.164	2.640	34	0.014*
Pair 7	Ashamed (before) —Ashamed (after)	0.538	1.303	0.256	0.012	1.065	2.107	34	0.045*
Pair 8	Nervous (before) —Nervous (after)	0.654	1.056	0.207	0.227	1.080	3.157	34	0.004**
Pair 9	Jittery (before) —Jittery (after)	0.269	0.962	0.189	-0.119	0.658	1.428	34	0.166
Pair 10	Afraid (before) —Afraid (after)	0.577	1.206	0.236	0.090	1.064	2.440	34	0.022*

*N* = 35.

*p < 0.05,

**p < 0.01,

***p < 0.001.

### 3.3 Results of children’s facial expressions analyzed using the google vision deep learning model

**FIGURE 8:**
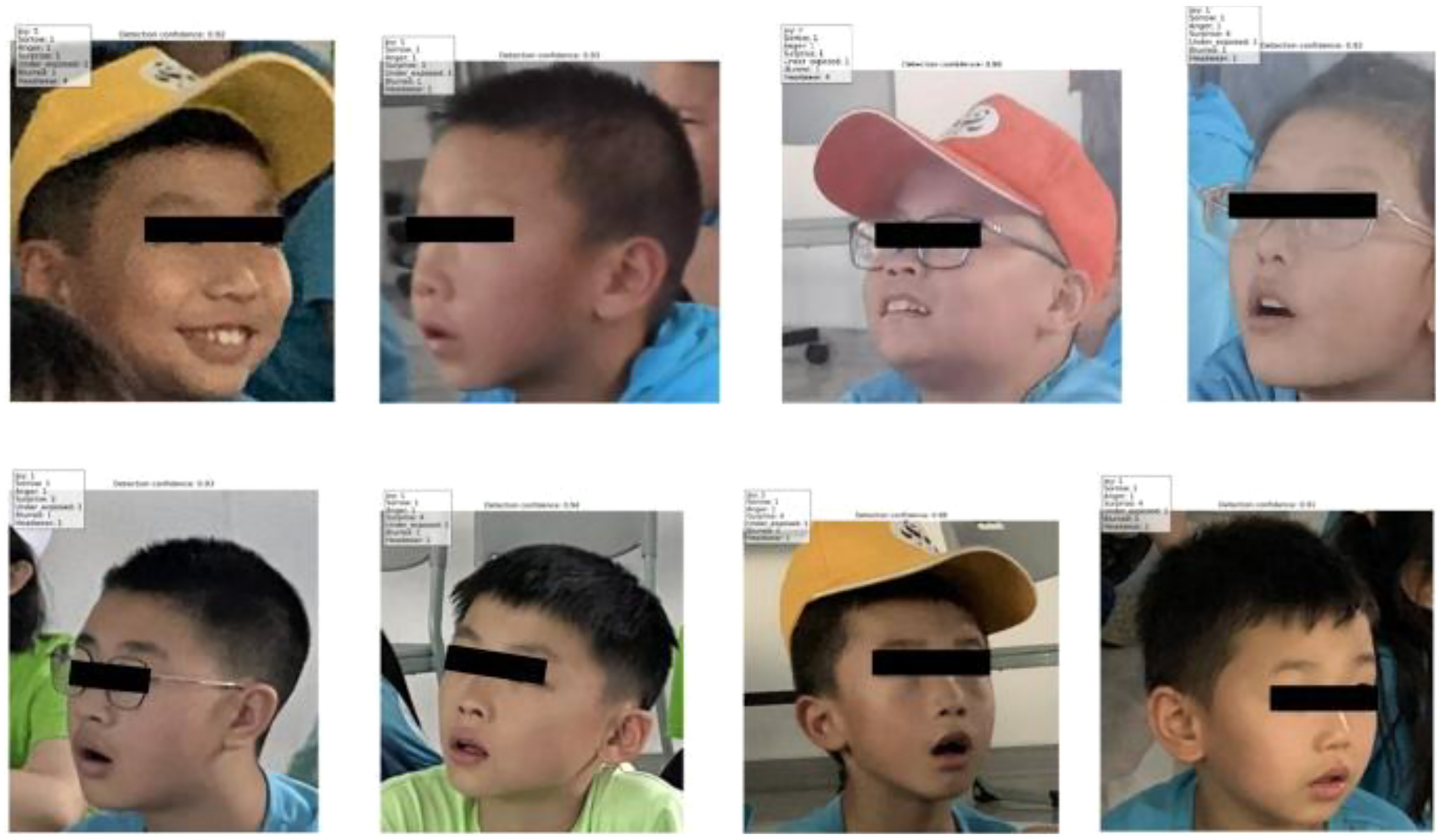
Emotion recognition results of the experiment 3 using the google vision deep learning model.

**FIGURE 9:**
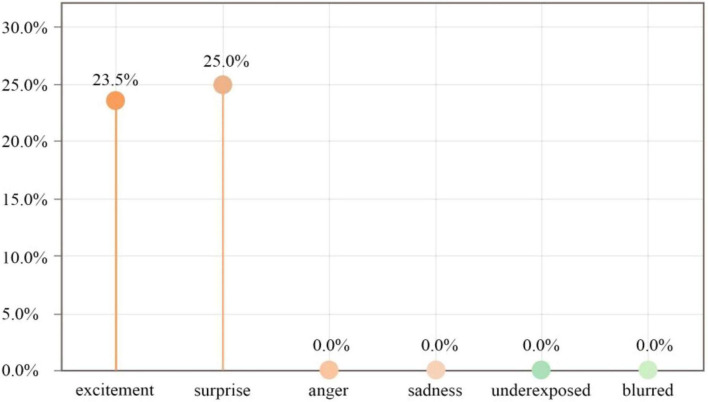
Facial emotion recognition results of the experiment 3 using the google vision deep learning model.

Using the facial emotion recognition functionality of the Google Vision Deep Learning Model, the facial expressions of children in Experiment 3 have been analyzed while the participants watched the “Flora and Fauna Species and Habitats of Giant Panda National Park” video (Figure 8). The results showed that expressions of surprise (curiosity/shock) had the highest proportion at 25%, followed by excitement (joy/happiness) at 23.5%, with no expressions of anger or sadness (Figure 9). The analysis indicated that participants could exhibit more positive emotional responses during symbolic contact with nature facilitated through immersive eye-care experiences, potentially yielding beneficial restorative outcomes.

**FIGURE 10:**
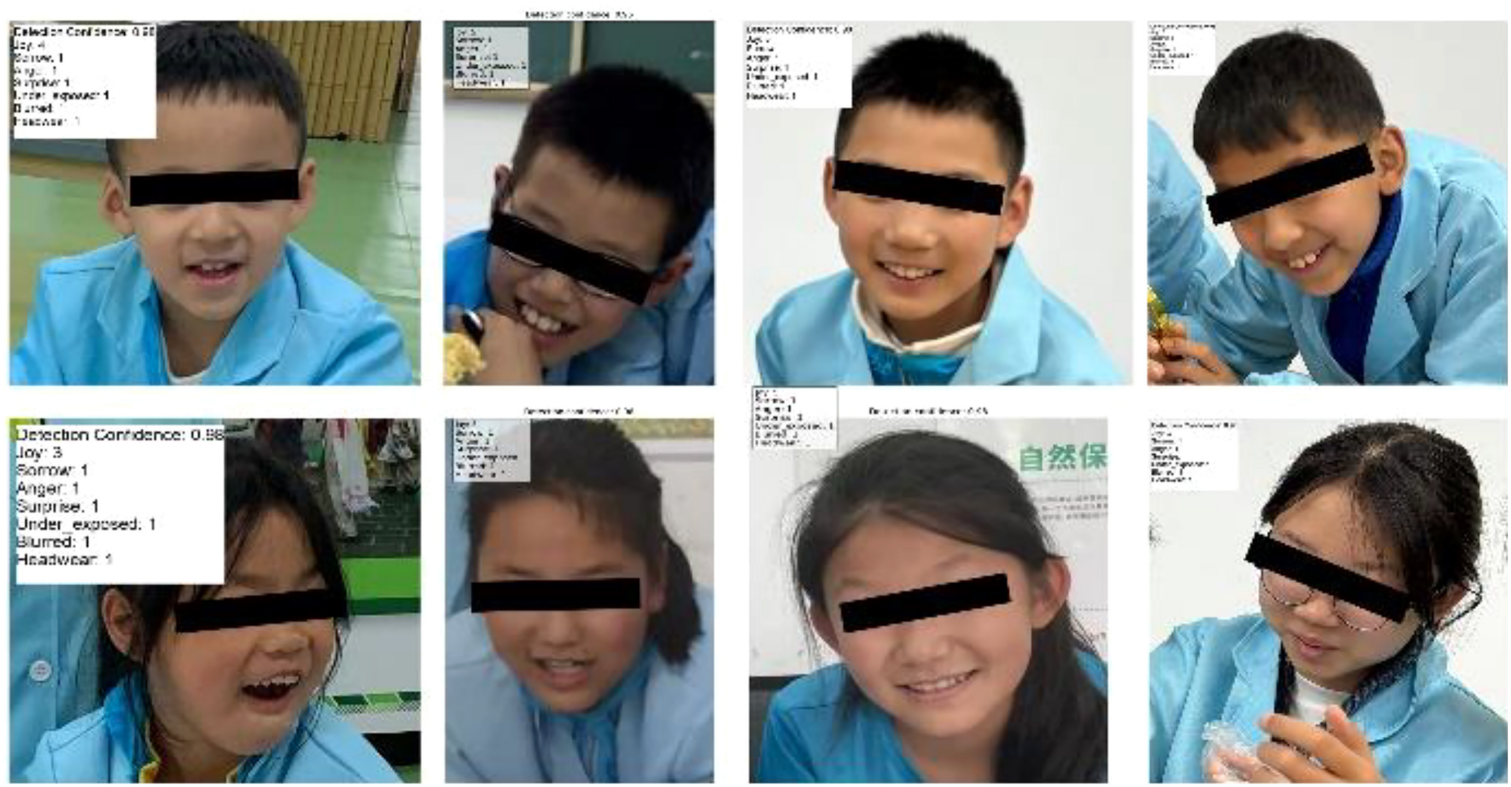
Facial emotion recognition results for the experiment 2 using the google vision deep learning model.

**FIGURE 11:**
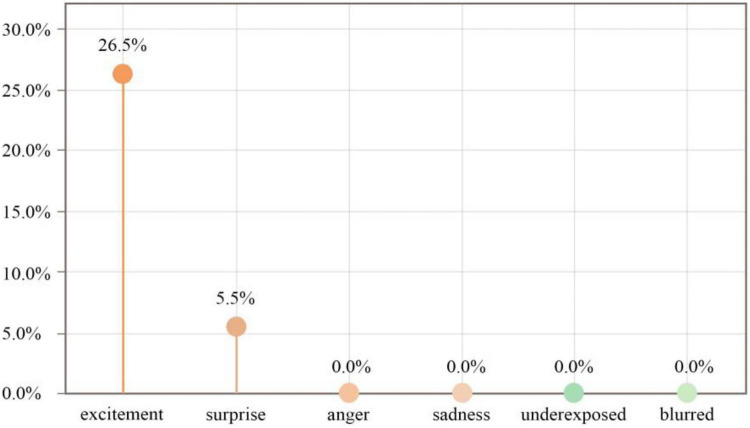
Facial emotion recognition results for the experiment 2 using the google vision deep learning model.

The results of the Experiment 2 (Figure 10) showed that excitement (joy/happiness) accounted for 26.5%, followed by surprise for 5.5%. Both anger and sadness had a 0% occurrence (Figure 11). This indicated that indirect nature education could elicit positive emotional effects in children, contributing to enhanced emotional experiences.

## 4 Discussion

### 4.1 Analysis of the immediate physiological restorativeness effects of different forms of nature education on children

Extensive research has explored the value of nature education for children (Sella et al., 2023). This study not only validates these benefits but also expands our understanding of the specific forms of children’s nature contact and their impacts on physiological and psychological restorative effects. Our findings indicate that compared to baseline levels before the experiments, both direct nature education and indirect nature education can significantly improve children’s immediate physiological restorativeness, while the former features multisensory interactions such as observation, experience, evaluation and exploration in the national park and indirect nature education focuses on scientific observation and creation in the Giant Panda National Park workshop. This finding provides more detailed evidence supporting the idea that nature education can help children achieve a sense of calmness and relaxation (Chawla, 2014; Korpela, 2002). Attention Restoration Theory (ART) states that direct attention can be restored by connection with natural environments (Kaplan, 1995). Related studies have reported that multisensory stimulation in preschool gardens significantly improves attention span for children aged from 0 to 6 (Whiren, 1995). Furthermore, our findings demonstrate that beyond direct contact, indirect interactions with nature also enhance children’s attention and relaxation, proving the restorative benefits of nature education through diverse engagement formats. Concurrently, the Stress Reduction Theory (SRT) proposes that nature contact alleviates stress by triggering involuntary physiological reactions, thereby inducing feelings of calmness (Ulrich et al., 1991). Previous studies have demonstrated that the process of manual creation fosters brain circuitry by allowing signals to move between neurons, creating new pathways and enriching experiences (Gulliksen, 2017). Consistent with SRT, our results suggest that both direct contact with relatively uncontrolled natural environments and indirect contact with nature under more structured, human-controlled settings, are significantly effective in enhancing children’s attention and relaxation.

Previous research has confirmed the positive effects of direct contact with nature on human health (Ives et al., 2018). However, it is unexpected that this study finds no significant difference between direct and indirect forms of nature contact in terms of their immediate physiological restorative effects on children’s attention and relaxation. Indirect contact with nature is equally important for children. It’s probably because during the “scientific observation and creation” activities in the workshop, children can experienced teamwork and mutual support, which are known to have broad positive effects on children’s physical and mental wellbeing, helping them by fostering a sense of safety and support (Mahoney et al., 2005; Graham et al., 2018). Additionally, it can be observed that endogenous opioids (EOPs), leading to hormonal changes, can be produced during hands-on creation and teamwork—such as discovering scientific wonders under the microscope, engaging in low-intensity organized scientific tasks, and experiencing the surprise, satisfaction and a sense of achievement when children witness their efforts come to fruition. Studies have shown that EOPs are widely distributed in the central nervous system (CNS) and can be found in cerebrospinal fluid, the pituitary gland, plasma, and many peripheral organs. Acute stress triggers the release of EOPs in the brain, spinal cord, and pituitary gland. EOPs help regulate stress responses by increasing pain threshold, raising core body temperature, enhancing exploratory and grooming behavior, alleviating anxiety, controlling pituitary secretions, and regulating peripheral organ activity during stress (Millan and Emrich, 1981). This suggests that hands-on activities in nature education play an important role in promoting children’s physical and psychological health.

Further analysis of the experimental data reveals that the increase in the average Theta wave value, associated with relaxation, is significantly higher in the direct nature contact compared with the indirect form. The primary distribution range of brainwave values for direct contact was also generally broader than those for indirect contact. Among the 35 participants, 24 of them exhibit at least one higher indicator among Theta, Alpha, or Beta wave value after direct contact compared with indirect contact. This finding indicates that the immediate physiological restorative effects of nature education through direct contact are significant and merit attention. Additionally, the distribution of both older and younger children, as well as both boys and girls, among the groups of, respectively, 24 and 11 participants, suggests that age and gender are not primary factors affecting the observed differences in immediate physiological restorativeness in children’s nature education.

### 4.2 Analysis of the immediate psychological restorativeness effects of different forms of nature education contact

Mackay and Neill (2010) reported that activities in nature were an important factor in promoting mental health. In our study, the paired *t*-test results of PANAS in Experiments 1 and 2 indicate that both direct and indirect forms of nature contact in nature education enhanced children’s positive emotions and alleviated their negative emotions, thereby improving mental health. A previous study suggests that natural settings may contribute to children’s perception of environmental restorativeness (Küçükaydın, 2024). Our research further confirms that compared with indirect and direct contact with nature resulted in significant improvements across all positive emotion indicators and marked decreases in all negative emotion indicators. In contrast, during indirect contact, the positive emotion indicator “attentive” shows no significant change before and after the experiment, and “jittery” among the negative emotions also shows no significant change. These findings suggest that direct contact with nature has a more comprehensive and significant effect on enhancing positive emotions and reducing negative emotions than indirect contact. Improvements in focused attention and reductions in restlessness can benefit children’s learning outcomes (Salla et al., 2016) and may lower the risk of developing Attention Deficit Hyperactivity Disorder (ADHD) (Markevych et al., 2018; Donovan et al., 2019). Additionally, neither form of nature contact led to a significant change in the negative emotion of “guilty.” Upon analysis, we found that children generally did not exhibit strong feelings of guilt before the experiments (with an average pre-experiment guilty score of 1.50), which likely accounts for the lack of a significant decrease in guilt emotions before and after the experiment.

### 4.3 Feasibility analysis of using the google vision deep learning model to measure children’s facial emotions in nature education

Previous research has demonstrated that deep learning models can serve as efficient tools for measuring emotions (Rathod et al., 2022). Building upon this foundation, our study integrates artificial intelligence with emotion recognition and nature education, suggesting that Google Vision Deep Learning Model may offer a potential tool for more objective and real-time analysis of children’s emotional responses in natural environments. The results of this study suggest that the Google Vision deep learning model ‘s facial emotion recognition functionality may be capable of identifying prominent emotions in children, such as joy and surprise, during various nature education activities. The detection of emotions like joy and surprise suggests that both the indirect “scientific observation and creation” form and the virtual immersive symbolic form of nature education can effectively elicit positive emotional responses in children. This provides new insights for broadening nature education methods and exploring additional approaches and technologies to study the benefits. However, this approach also faces certain limitations. One challenge lies in the scarcity of an excellent illustrative dataset for training in sufficient quality and quantity, which may lead to imbalances in gender, age, face color and cross-cultural difference. Furthermore, children’s facial emotions can be fleeting and highly variable, making it difficult for models alone to capture subtle emotional shifts. Future research could enhance credibility by incorporating manually annotated validation.

This study offers flexible options for future nature education practices. When adolescents can not engage directly with natural environments due to time constraints, limited access, or fear of biological encounters, indirect and symbolic forms of nature education remain meaningful. For example, it is significant to establish nature workshops in cities and enhance children’s interaction with nature in botanical gardens, zoos and community parks, as well as integrate nature education into science, geography and biology curricula in schools by introducing hands-on courses like ecological planting and insect observation. There also are some ways such as by creating school gardens, breeding areas, and ecological experiment fields to provide on-campus nature experiences; by incorporating art, literature, and technology through painting, writing and experiments to foster a multi-dimensional appreciation of nature; by developing nature education apps, VR/AR virtual explorations, and interactive science games with drones, smart monitoring stations, and remote cameras to enable real-time ecological observation. These activities, including scientific observation, hands-on creation, and teamwork, can provide children with feelings of safety, support, surprise, satisfaction and achievement. Additionally, brief virtual nature experiences can similarly enhance environmental awareness and foster a connection with nature (Owens and Bunce, 2023; Litleskare et al., 2020). However, with proper conditions, the physiological and psychological restorative benefits of direct contact with nature for children remain highly valuable and should be prioritized (Yu et al., 2020).

## 5 Conclusion

This study confirms that contact with nature has an immediate restorative effect on children, enhancing both attention and relaxation. Both direct and indirect forms of nature contact provide significant immediate physiological benefits by improving attention and relaxation, promoting positive emotional development and reducing negative emotions. Notably, direct form of nature contact appears particularly impactful. The exploratory application of Google Vision computer model for detecting children’s prominent emotions during various nature education activities shows preliminary feasibility, suggesting that indirect and virtual immersive symbolic forms of nature education may also elicit positive emotional responses of children. One important consideration is the potential limitations of Google Vision’s deep learning model in facial emotion detection, particularly its sensitivity to subtle or complex emotions in children and the influence of cultural context on emotional expressions. For detecting nuanced emotional changes in multicultural settings, cross-validating Google Vision’s output with human-coded facial expressions can enhance the completeness and reliability of emotion classification.

The study cautiously considers variables such as children’s age, gender and experiment duration to ensure data representativeness. However, certain limitations remain, including potential variability in outcomes based on children’s family backgrounds and their frequency of exposure to green spaces. For instance, children with different levels of prior exposure to nature may respond differently to nature education in national parks. Additionally, due to the different levels of objectivity among the three experiments, comparison across varying conditions are not feasible, potentially introducing bias. Given this study’s consideration for children’s visual health, the results from Experiment 3 cannot conclusively determine whether longer viewing times yield consistent emotional effects in children.

In terms of activities chosen for indirect nature contact, the experimental activities include making food for giant pandas (steamed buns), observing their feces, and creating plant specimens. Future research should explore more varieties of nature education activities over extended periods to further assess the potential of national parks as sites for nature education. It is particularly significant for children, as it may help mitigate “Nature-Deficit Disorder” and enhance their overall health and wellbeing.

Additionally, our study focuses on the immediate physiological and psychological benefits of different forms of nature education. Using a basic pre-and-post-experimental design, it is assessed whether the intervention can lead to changes in physiological and psychological indicators. Notably, our hypothesis was supported, confirming the immediate benefits of nature education. Thus, the findings can serve as exploratory evidence. Admittedly, this study does not include a control group (e.g., children without participating in any nature education activity), which may limit the causal attribution of the observed changes to the interventions. Furthermore, the sample size of 35 children, while adequate for preliminary paired analyses, is relatively small and may not fully represent broader population. In this study, the absence of a control group and the relatively small sample size are explicitly and clearly important methodological limitations. Future studies should consider larger and more diverse samples, as well as employ more complex environmental designs, such as randomized controlled trials, to further validate the significance of nature education, particularly in comparison with urban control groups, and to explore its role in urban education. Moreover, future research could investigate whether the restorative benefits of nature contact vary across different age groups.

This study also aims to integrate more forms of nature education into future practices, fostering children’s appreciation of nature and biodiversity while instilling a strong sense of responsibility for conservation. It seeks to raise concerns of global environmental issues, cultivate environmental literacy and promote conservation awareness. Additionally, nature education helps shape children’s moral values related to conservation, strengthen their sense of connection to their community and nation, and cultivate future citizens with a strong sense of social responsibility and good adaptive capacity.

## Data Availability

The original contributions presented in the study are included in the article/Supplementary material, further inquiries can be directed to the corresponding author.

## References

[B1] Bagot K. L. Allen F. C. L. Toukhsati S. (2015). Perceived restorativeness of children’s school playground environments: Nature, playground features and play period experiences. *J. Environ. Psychol.* 41 1–9. 10.1016/j.jenvp.2014.11.005

[B2] Beatley T. (2011). *Biophilic Cities: Integrating Nature Into Urban Design and Planning.* Washington, DC: Island Press.

[B3] Burdette H. L. Whitaker R. C. (2005). Resurrecting free play in young children. *Arch. Pediatrics Adolescent Med.* 159:46. 10.1001/archpedi.159.1.46 15630057

[B4] Ç,etinkaya Ç (2013). Sakarya science and art center nature education program. *J. Environ. Protect. Ecol.* 14 1317–1324.

[B5] Cetinkaya C. (2013). Creative nature education program for gifted and talented students. *Anthropologist* 16 691–699. 10.1080/09720073.2013.11891395

[B6] Chawla L. (2014). *Children’s Engagement with the Natural World as a Ground for Healing.* Dordrecht: Springer Netherlands, 10.1007/978-90-481-9947-1_8

[B7] Cheng J. C. H. Monroe M. (2012). Connection to nature: Children’s affective attitude toward nature. *Environ. Behav.* 44 31–49. 10.1177/0013916510385082

[B8] Clements R. (2004). An investigation of the status of outdoor play. *Contemp. Issues Early Childhood* 5 68–80. 10.2304/ciec.2004.5.1.10

[B9] Collado S. Staats H. Corraliza J. A. (2013). Experiencing nature in children’s summer camps: Affective, cognitive and behavioural consequences. *J. Environ. Psychol.* 33 37–44. 10.1016/j.jenvp.2012.08.002

[B10] Dale R. G. Powell R. B. Stern M. J. Garst B. A. (2020). Influence of the natural setting on environmental education outcomes. *Environ. Educ. Res.* 26 613–631. 10.1080/13504622.2020.1738346

[B11] Dalvi C. Rathod M. Patil S. Gite S. Kotecha K. (2021). A survey of AI-Based facial emotion recognition: Features, ML DL techniques, age-wise datasets and future directions. *IEEE Access* 9 165806–165840. 10.1109/ACCESS.2021.3131733 25079929

[B12] Donovan G. H. Michael Y. L. Gatziolis D. Mannetje A. Douwes P. J. (2019). Association between exposure to the natural environment, rurality, and attention-deficit hyperactivity disorder in children in New Zealand: A linkage study. *Lancet Planetary Health* 3 e226–e234. 10.1016/S2542-5196(19)30070-1 31128768

[B13] Dopko R. L. Capaldi C. A. Zelenski J. M. (2019). The psychological and social benefits of a nature experience for children: A preliminary investigation. *J. Environ. Psychol.* 63 134–138. 10.1016/j.jenvp.2019.05.002

[B14] Dudley N. Higgins-Zogib L. H. Hockings M. MacKinnon K. Sandwith T. Stolton S. (2011). National parks with benefits: How protecting the planet’s biodiversity also provides ecosystem services. *Solutions* 2 87–95.

[B15] Fachner J. Gold C. Erkkilä J. (2013). Music therapy modulates fronto-temporal activity in Rest-EEG in depressed clients. *Brain Topography* 26 338–354. 10.1007/s10548-012-0254-x 22983820

[B16] Fu W. Z. (2021). A brief discussion about children’s nature-deficit disorder: Based on analysis of related literature in British academia. *J. Jiangsu Second Normal Univ.* 37 37–44+124.

[B17] Fyfe-Johnson A. L. Hazlehurst M. F. Perrins S. P. Bratman G. N. Thomas R. Garrett K. A. et al. (2021). Nature contact and Children’s health: A systematic review. *Pediatrics* 147 50–52. 10.1542/peds.2020-049155 34588297

[B18] Graham A. Truscott J. Simmons C. Anderson D. Thomas N. (2018). Exploring student participation across different arenas of school life. *Br. Educ. Res. J.* 44 1029–1046. 10.1002/berj.3477

[B19] Gulliksen M. S. (2017). Making matters? Unpacking the role of practical aesthetic making activities in the general education through the theoretical lens of embodied learning. *Cogent Educ.* 4:1415108. 10.1080/2331186x.2017.1415108

[B20] Harris F. (2018). Outdoor learning spaces: The case of forest school. *Area* 50 222–231. 10.1111/area.12360

[B21] Hartig T. (2004). *Restorative Environments.* San Diego, CA: Academic Press.

[B22] Huang X. (2020). The children-nature relationship: Contact, cognization and emotion. *Hum. Geography* 35 9–17+75.

[B23] Ives C. D. Abson D. J. Von Wehrden H. Dorninger C. Klaniecki K. Fischer J. (2018). Reconnecting with nature for sustainability. *Sustainabil. Sci.* 13 1389–1397. 10.1007/s11625-018-0542-9 30220917 PMC6132401

[B24] Juster F. T. Ono H. Stafford F. P. (2004). *Changing Times of American Youth: 1981-2003.* Ann Arbor, MI: Institute for Social Research, University of Michigan.

[B25] Kahn P. H. Jr. Kellert S. R. (2002). *Children and Nature: Psychological, Sociocultural, and Evolutionary Investigations.* Cambridge, MA: MIT Press, 10.7551/mitpress/1807.001.0001

[B26] Kaplan S. (1995). The restorative benefits of nature: Toward an integrative framework. *J. Environ. Psychol.* 15 169–182. 10.1016/0272-4944(95)90001-2

[B27] Klimesch W. (1999). EEG alpha and theta oscillations reflect cognitive and memory performance: A review and analysis. *Brain Res. Rev.* 29 169–195. 10.1016/S0165-0173(98)00056-3 10209231

[B28] Korpela K. (2002). “Children’s Environment,” in *Handbook of Environmental Psychology*, Vol. John Wiley & Sons eds Bechtel R. B. Churchman A. (New York, NY).

[B29] Küçükaydın M. A. (2024). Understanding connection to nature in Turkish middle school children: Personal factors and Nature’s restorative effect. *J. Environ. Psychol.* 98:102393. 10.1016/j.jenvp.2024.102393

[B30] Labintah S. Shinozaki M. (2014). Children drawing: Interpreting school-group student’s learning and preferences in environmental education program at TanjungPiai National Park, Johor Malaysia. *Procedia - Soc. Behav. Sci.* 116 3765–3770. 10.1016/j.sbspro.2014.01.838

[B31] Lee B. G. Lee B. L. Chung W. Y. (2014). Mobile healthcare for automatic driving sleep-onset detection using wavelet-based EEG and respiration signals. *Sensors* 14 17915–17936. 10.1016/S0165-0173(98)00056-3 25264954 PMC4239919

[B32] Li S. L. (2017). Nature-based education for children in American early childhood programs and its enlightenment. *Internatl. Comp. Educ.* 39 97–105.

[B33] Li X. C. Sun X. L. Hong X. J. (2023). Analysis on the nature education system of the Tropical Rain Forest National Park in Hainan from the Third Party Perspective. *World J. For.* 12 27–35. 10.12677/WJF.2023.121005

[B34] Li Y. Yu L. Dou Y. Q. (2022). Path to improve natural education function of China’s National Parks based on international experience. *World For. Res.* 35 113–118.

[B35] Linzmayer C. D. Halpenny E. A. (2014). ‘I might know when I’m an adult’: Making sense of children’s relationships with nature. *Children’s Geograph.* 12 412–428. 10.1080/14733285.2013.821262

[B36] Litleskare S. MacIntyre T. E. Calogiuri G. (2020). Enable, reconnect and augment: A new era of virtual nature research and application. *Intern. J. Environ. Res. Public Health* 17:1738. 10.3390/ijerph17051738 32155911 PMC7084893

[B37] Louv R. (2008). *Last Child in the Woods: Saving Our Children from Nature-deficit Disorder.* New York, NY: Algonquin books.

[B38] Mackay G. J. Neill J. T. (2010). The effect of ‘green exercise’ on state anxiety and the role of exercise duration, intensity, and greenness: A quasi-experimental study. *Psychol. Sport Exerc.* 11 238–245.

[B39] Mahoney J. L. Larson R. W. Eccles J. S. (2005). *Organized Activities as Contexts of Development: Extracurricular Activities, After School and Community Programs.* New York, NY: Psychology Press, 10.4324/9781410612748

[B40] Markevych I. Tesch F. Datzmann T. Romanos M. Schmitt J. Heinrich J. (2018). Outdoor air pollution, greenspace, and incidence of ADHD: A semi-individual study. *Sci. Total Environ.* 642 1362–1368. 10.1016/j.scitotenv.2018.06.167 30045516

[B41] Martin L. White M. P. Hunt A. Richardson M. Pahl S. Burt J. (2020). Nature contact, nature connectedness and associations with health, wellbeing and pro-environmental behaviours. *J. Environ. Psychol.* 68:101389. 10.1016/j.jenvp.2020.101389

[B42] Medvedev O. N. Roemer A. Krägeloh C. U. Sandham M. H. Sigert R. J. (2023). Enhancing the precision of the Positive and Negative Affect Schedule (PANAS) using Rasch analysis. *Curr. Psychol.* 42 1554–1563. 10.1007/s12144-021-01556-3

[B43] Millan M. J. Emrich H. M. (1981). Endorphinergic systems and the response to stress. *Psychotherapy Psychosomat.* 36 43–56. 10.1159/000287525 6276913

[B44] Novikova E. Pic A. Han M. (2024). Language use in indoor and outdoor settings among children in a nature-based preschool. *Environ. Educ. Res.* 30 138–152. 10.1080/13504622.2023.2246688

[B45] Otto S. Pensini P. (2017). Nature-based environmental education of children: Environmental knowledge and connectedness to nature, together, are related to ecological behaviour. *Global Environ. Change* 47 88–94. 10.1016/j.gloenvcha.2017.09.009

[B46] Owens M. Bunce H. (2023). The effect of brief exposure to virtual nature on mental wellbeing in adolescents. *Sci. Rep.* 13:17769. 10.1038/s41598-023-44717-z 37853074 PMC10584913

[B47] Rathod M. Dalvi C. Kaur K. Patil S. Gite S. Kamat P. et al. (2022). Kids’ emotion recognition using various deep-learning models with explainable AI. *Sensors* 22:8066. 10.3390/s22208066 36298415 PMC9607169

[B48] Salla J. Michel G. Pingault J. B. Lacourse E. Paquin S. Galéra C. et al. (2016). Childhood trajectories of inattention-hyperactivity and academic achievement at 12 years. *Eur. Child Adolescent Psychiatry* 25 1195–1206. 10.1007/s00787-016-0843-4 27017347

[B49] Schacter D. L. (1977). EEG theta waves and psychological phenomena: A review and analysis. *Biol. Psychol.* 5 47–82. 10.1016/0301-0511(77)90028-X 193587

[B50] Schaffer H. R. (2004). *Introducing Child Psychology.* Hoboken, NJ: Blackwell Publishing.

[B51] Sella E. Bolognesi M. Bergamini E. Mason L. Pazzaglia F. (2023). Psychological benefits of attending forest school for preschool children: A systematic review. *Educ. Psychol. Rev.* 35:29. 10.1007/s10648-023-09750-4

[B52] Sprague N. L. Bancalari P. Karim W. Siddiq S. (2022). Growing up green: A systematic review of the influence of greenspace on youth development and health outcomes. *J. Exposure Sci. Environ. Epidemiol.* 32 660–681. 10.1038/s41370-022-00445-6 35614136 PMC9482936

[B53] Sprague N. Berrigan D. Ekenga C. C. (2020). An analysis of the educational and health-related benefits of nature-based environmental education in low-income black and hispanic children. *Health Equity* 4 198–210. 10.1089/heq.2019.0118 32440617 PMC7241057

[B54] Tang F. L. (2018). Reflections on the construction of national park system with Chinese characteristics. *For. Construct.* 5 86–96.

[B55] Ulrich R. S. Simons R. F. Losito B. D. Fiorito E. Miles M. A. Zelson M. (1991). Stress recovery during exposure to natural and urban environments. *J. Environ. Psychol.* 11 201–230. 10.1016/s0272-4944(05)80184-7

[B56] Uzun F. V. Keles O. (2012). The effects of nature education project on the environmental awareness and behavior. *Procedia – Soc. Behav. Sci.* 46 2912–2916. 10.1016/j.sbspro.2012.05.588

[B57] van Dijk–Wesselius J. E. Maas J. Hovinga D. van Vugt M. van den Berg A. E. (2018). The impact of greening schoolyards on the appreciation, and physical, cognitive and social-emotional well-being of schoolchildren: A prospective intervention study. *Landscape Urban Plann.* 180 15–26. 10.1016/j.landurbplan.2018.08.003

[B58] Wang Z. Y. Shi L. (2020). Literature review on natural education in China: Based on the statistical analysis of Bibexcel software. *For. Econ.* 42 83–92.

[B59] Watson D. Clark L. A. Tellegen A. (1988). Development and validation of brief measures of positive and negative affect: The PANAS scales. *J. Personal. Soc. Psychol.* 54 1063–1070. 10.1037//0022-3514.54.6.1063 3397865

[B60] Whiren A. P. (1995). Planning a garden from a child’s perspective. *Children’s Environ.* 12 250–255.

[B61] Yao X. Y. Zhang Y. (2023). Value co-creation mechanism of tourists participation in nature education in Giant Panda National Park. *J. Chin. Urban For.* 21 102–108.

[B62] Yong Y. (2024). Study on the nature education spatial suitability analysis of Gutianshan area of Qianjiangyuan National Park. *Nat. Protected Areas* 4 12–25. 10.12335/2096-8981.2024050601

[B63] Yu C. P. Lee H. Y. Lu W. H. Huang Y. C. Browning M. H. E. M. (2020). Restorative effects of virtual natural settings on middle-aged and elderly adults. *Urban For. Urban Greening* 56:126863. 10.1016/j.ufug.2020.126863

[B64] Yu X. R. Qiu L. Yu H. L. (2023). Research on promoting community environmental responsibility behaviors in the National Park through nature education: A case study in the Three–River–Source. *Nat. Protected Areas* 3 91–101.

[B65] Yu X. W. Du C. L. (2022). Research on the intervention model of urban community garden for children with Nature-Deficit Disorder. *J. Hum. Settlements West China* 37 35–39.

