# Supplementary material for: A multidimensional exploration of the immediate physiological and psychological restorativeness of nature education for children in Giant Panda National Park
Source: Front Psychol. 2026 Jun 26;17:1551296. doi: 10.3389/fpsyg.2026.1551296 (PMC13349400; doi:10.3389/fpsyg.2026.1551296)
Supplement: Supplementary file 1 [file Supplementary_file_1.docx]

**Appendix Table.** Positive and Negative Affect Schedule (PANAS)

| Number | PANAS | Very slightly or not at all  1 | A little  2 | Moderately  3 | Quite a bit  4 | Extremely  5 |
| --- | --- | --- | --- | --- | --- | --- |
| 1 | Interested |  |  |  |  |  |
| 2 | Distressed |  |  |  |  |  |
| 3 | Excited |  |  |  |  |  |
| 4 | Upset |  |  |  |  |  |
| 5 | Strong |  |  |  |  |  |
| 6 | Guilty |  |  |  |  |  |
| 7 | Scared |  |  |  |  |  |
| 8 | Hostile |  |  |  |  |  |
| 9 | Enthusiastic |  |  |  |  |  |
| 10 | Proud |  |  |  |  |  |
| 11 | Irritable |  |  |  |  |  |
| 12 | Alert |  |  |  |  |  |
| 13 | Ashamed |  |  |  |  |  |
| 14 | Inspired |  |  |  |  |  |
| 15 | Nervous |  |  |  |  |  |
| 16 | Determined |  |  |  |  |  |
| 17 | Attentive |  |  |  |  |  |
| 18 | Jittery |  |  |  |  |  |
| 19 | Active |  |  |  |  |  |
| 20 | Afraid |  |  |  |  |  |
